# Prediction of circRNA–Disease Associations via Graph Isomorphism Transformer and Dual-Stream Neural Predictor

**DOI:** 10.3390/biom15020234

**Published:** 2025-02-06

**Authors:** Hongchan Li, Yuchao Qian, Zhongchuan Sun, Haodong Zhu

**Affiliations:** School of Computer Science and Technology, Zhengzhou University of Light Industry, Zhengzhou 450000, China; 2011017@zzuli.edu.cn (H.L.); 332207050674@zzuli.edu.cn (Y.Q.); zcsun@zzuli.edu.cn (Z.S.)

**Keywords:** circRNA–disease associations, transformer, graph isomorphism network, knowledge representation learning

## Abstract

Circular RNAs (circRNAs) have attracted increasing attention for their roles in human diseases, making the prediction of circRNA–disease associations (CDAs) a critical research area for advancing disease diagnosis and treatment. However, traditional experimental methods for exploring CDAs are time-consuming and resource-intensive, while existing computational models often struggle with the sparsity of CDA data and fail to uncover potential associations effectively. To address these challenges, we propose a novel CDA prediction method named the Graph Isomorphism Transformer with Dual-Stream Neural Predictor (GIT-DSP), which leverages knowledge graph technology to address data sparsity and predict CDAs more effectively. Specifically, the model incorporates multiple associations between circRNAs, diseases, and other non-coding RNAs (e.g., lncRNAs, and miRNAs) to construct a multi-source heterogeneous knowledge graph, thereby expanding the scope of CDA exploration. Subsequently, a Graph Isomorphism Transformer model is proposed to fully exploit both local and global association information within the knowledge graph, enabling deeper insights into potential CDAs. Furthermore, a Dual-Stream Neural Predictor is introduced to accurately predict complex circRNA–disease associations in the knowledge graph by integrating dual-stream predictive features. Experimental results demonstrate that GIT-DSP outperforms existing state-of-the-art models, offering valuable insights for precision medicine and disease-related research.

## 1. Introduction

Circular RNA (circRNA) is an emerging non-coding RNA known for its unique circular and highly stable structure [1]. In recent years, circRNA has attracted considerable interest due to its significance in studying human disease mechanisms, such as cancer, neurological diseases, and cardiovascular diseases. The pervasive presence and different roles of circRNA make them key regulators in the gene expression regulatory network. For example, circ*MTA2* was found to promote gastric cancer progression by interacting with UCHL3 to inhibit *MTA2* ubiquitination and stabilize its expression [2]. In addition, hsa_circ_002048 was found to regulate miRNAs and *AP2M1* expression, impairing autophagy and promoting β-amyloid deposition, which contributed to Alzheimer’s disease progression [3]. Therefore, comprehensive studies of circRNA–disease associations (CDAs) can provide new insights and strategies for understanding disease pathogenesis and significantly influence the treatment and prevention of human diseases [4]. However, conventional experimental approaches for CDA discovery face significant limitations due to their reliance on labor-intensive protocols, sophisticated equipment, and the inherent complexity of circRNA biology [5]. To address these challenges, researchers have proposed a variety of computational methods for CDA prediction. These methods are typically classified into three categories, including information-propagation-based, traditional machine-learning-based, and deep-learning-based [6].

Information-propagation-based methods typically use disease semantic similarity, circRNA functional similarity, and circRNA–target gene associations to construct heterogeneous networks for CDA prediction. To put it simply, these methods are like constructing a complex social network where each node represents a disease or a circRNA, and the connections between nodes indicate their similarities or associations. By analyzing this network, we can uncover potential links, aiding in the prediction of relationships between circRNAs and diseases. For example, Fan et al. [7] and Deng et al. [8] proposed the KATZHCDA and KATZCPDA models, respectively, to infer potential CDAs using the KATZ algorithm. Subsequently, Vural et al. [9] predicted CDAs based on fused similarity matrices combined with a random walk with restart (RWR) algorithm. Wang et al. [10] designed a method called PreCDA to infer CDAs using a graph recommendation algorithm. Recently, Zhang et al. [11] used the linear neighborhood label propagation approach CDLNLP to apply an LP algorithm to the computed similarity matrix for prediction. Similarly, Ge et al. [12] employed the locally constrained coding method LLCDC to achieve the prediction of potential CDAs. Although such methods place little strain on computer resources, they are highly dependent on known biological networks (e.g., gene regulatory networks). Additionally, the prediction results can be severely affected when the network knowledge is incomplete or inaccurate.

Traditional machine-learning-based approaches have rapidly developed in the field of CDA prediction. These approaches often rely on well-established algorithms and models to analyze data and make predictions. For example, Lei et al. [13] were the first to design the GBDTCDA method, which uses the gradient boosting decision tree algorithm and various data as integrated features for CDA prediction. Lei et al. [14] then introduced RWRKNN, a method that used the random walk with restart algorithm to construct feature vectors for circRNA and disease, followed by the k-nearest neighbor algorithm for CDA prediction. Wei et al. [15] considered the excellent performance of matrix factorization (MF) in handling noisy data and proposed the ICIRCDA-MF method for CDA prediction, correcting false-negative associations. Recently, Peng et al. [16] used a robust non-negative MF method combined with the LP algorithm (RNMFLP) to predict potential CDAs. These methods typically provide relatively accurate prediction results but require manual design and selection of features and may require retraining when dealing with new circRNA–disease associations in complex biological networks.

Deep-learning-based methods have been highly successful in predicting CDA tasks. In simpler terms, these methods use advanced algorithms to automatically learn intricate patterns and associations from the data, and have been very effective in predicting how circRNAs might be associated with diseases. Initially, Lu et al. [17] introduced the DMFCDA model, which utilizes a multi-layer neural network (NN) to learn potential features for predicting CDAs. Subsequently, Lan et al. [18] improved the graph convolutional neural network and proposed the IGNSCDA method, which also used negative sampling to reduce the effect of potential noise. After that, they designed a model named KGANCDA [19] incorporating knowledge graph attention networks, which innovatively constructed a knowledge graph to capture potential higher-order information and thus predict potential CDAs more effectively. Unlike Lan, Niu et al. [20] developed GMNN2CD, a prediction approach that uses variational inference and Markov neural networks to predict CDAs after constructing integrated similarity descriptors. Recently, Wu et al. [21] introduced the KGETCDA method, which uses a Transformer encoder to obtain embeddings and then employs a graph convolutional network (GCN) for updating and aggregating knowledge graph information. While such methods have been well developed for feature extraction, they have limitations in distinguishing differences between features and dealing with complex non-linear associations (referring to intricate association patterns between circRNAs and diseases learned through embeddings).

Although current approaches have achieved notable success in CDA prediction, they are still constrained by two primary limitations. Firstly, existing methods struggle to address the sparsity of CDA data, resulting in suboptimal models for CDA prediction. Secondly, current models are insufficient in exploring diverse higher-order information and fail to capture the complex non-linear associations between circRNAs and diseases. In short, it is difficult to mine the potential associations in circRNAs and diseases.

To address the above limitations, this paper introduces a novel computational approach named the Graph Isomorphism Transformer with Dual-Stream Neural Predictor (GIT-DSP) to address data sparsity and uncover potential high-order circRNA–disease associations. In simple terms, we innovatively integrated the Graph Isomorphism Network into the Transformer architecture to form the Graph Isomorphism Transformer module, which learns higher-order knowledge representations. Then, we improved the traditional classifier and designed a new predictor called the Dual-Stream Neural Predictor to predict potential circRNA–disease associations. Specifically, GIT-DSP integrates a variety of associations among diseases, circRNAs, lncRNAs, and miRNAs in multi-source datasets. Then, the information on disease semantic similarity, circRNA Gaussian interaction profile kernel similarity, and circRNA functional similarity are fused to construct an integrative knowledge graph, which alleviates the data sparsity problem. Next, we propose the Graph Isomorphism Transformer to derive high-quality knowledge representations and capture both local and global association information in the knowledge graph. Finally, a Dual-Stream Neural Predictor is employed to accurately predict potential circRNA–disease associations by integrating dual-stream predictive features. Experimental results show that GIT-DSP outperforms other state-of-the-art (SOTA) models.

The main contributions are as follows:

- We propose an efficient knowledge representation learning method using a Graph Isomorphism Transformer, which fully captures local and global circRNA–disease associations within a multi-source heterogeneous knowledge graph, enabling higher-order associative knowledge representation and addressing the data sparsity problem;
- We introduce a Dual-Stream Neural Predictor specifically designed for circRNA–disease association prediction, which captures complex non-linear associations and significantly improves prediction accuracy and computational efficiency;
- Extensive experiments have shown that the GIT-DSP model is superior in CDA prediction and has great potential in the fields of non-coding RNA (ncRNA) and protein association prediction.

The remaining parts of this paper are developed as follows: Section 2 covers dataset selection and the GIT-DSP model proposal. Section 3 discusses the evaluation criteria, parameter settings, performance comparison, ablation studies, and specific case studies. Section 4 covers conclusions and future work on GIT-DSP.

## 2. Materials and Methods

**Figure 1:**
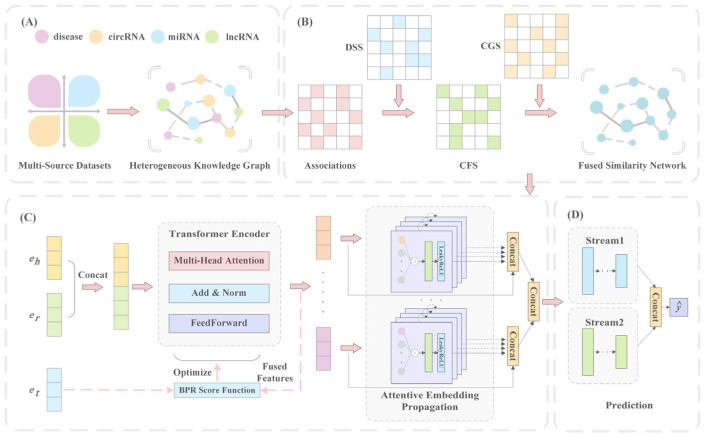
Flowchart of GIT-DSP. (**A**) Construct the CDA knowledge graph with multi-source heterogeneous datasets. (**B**) Construct a fused similarity network based on the association matrix. (**C**) Generate high-quality embeddings through attention-based information propagation with the Graph Isomorphism Transformer. (**D**) Predict CDAs.

In this section, we propose the GIT-DSP model. GIT-DSP effectively extracts complex higher-order information from multi-source heterogeneous data with outstanding representation and prediction abilities. Figure 1 shows the model framework, including four key parts: knowledge graph construction, similarity measure, knowledge representation, and model prediction. First, a knowledge graph covering disease, circRNA, lncRNA, miRNA, and their associations is constructed. Next, a fusion similarity network is formed by integrating disease semantic similarity (DSS), circRNA gaussian interaction profile kernel similarity (CGS), and circRNA functional similarity (CFS). Using the Transformer backbone, the graph isomorphism network is designed to propagate and aggregate information, capturing local and global associations in the knowledge graph. Finally, a Dual-Stream Neural Predictor is proposed to predict affinity scores for CDAs, enhancing the recognition of non-linear associations among CDAs.

### 2.1. Datasets

In this study, we constructed three datasets based on previous work [19,21]. Dataset 1 comprised non-cancer-related data, including 79 diseases, 330 circRNAs, 404 lncRNAs, and 265 miRNAs. Additionally, 1327 associations were extracted from it. Dataset 2 used cancer-related data to further validate the robustness of the GIT-DSP model. After de-duplication, we obtained 62 diseases, 514 circRNAs, 666 lncRNAs, and 573 miRNAs, along with a total of 3509 pieces of associations. In addition, we incorporated an ultra-large multi-source heterogeneous dataset, Dataset 3, which contains information from 13 public databases, including 190 diseases, 561 circRNAs, 658 miRNAs, 1043 lncRNAs, and 25,468 associations. The incorporation of this dataset will allow for more in-depth exploration and understanding of the molecular mechanisms of various diseases and a more rigorous evaluation of the performance of GIT-DSP. Table 1 shows the summary data for the three datasets.

**Figure 2:**
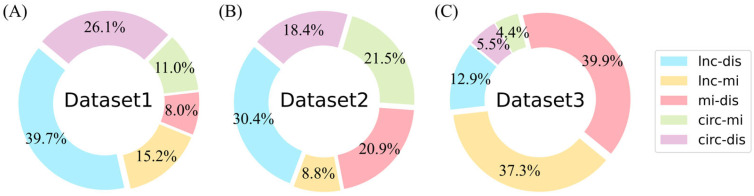
Visualization of datasets. (**A**–**C**) represent the association distributions of Dataset 1, Dataset 2, and Dataset 3, respectively. This contains five associations (lnc-dis (lncRNA–disease), lnc-mi (lncRNA–miRNA), mi-dis (miRNA–disease), circ-mi (circRNA–miRNA), and circ-dis (circRNA–disease)).

To provide a more intuitive description of the data distribution, the three datasets were visualized. Figure 2A–C show the distribution of the five types of associations in Dataset 1, Dataset 2, and Dataset 3, respectively. There is a notable difference in the distribution of the number of associations in each category. Notably, circRNA–disease associations account for a relatively low number, posing a challenge for CDA prediction.

**Figure 3:**
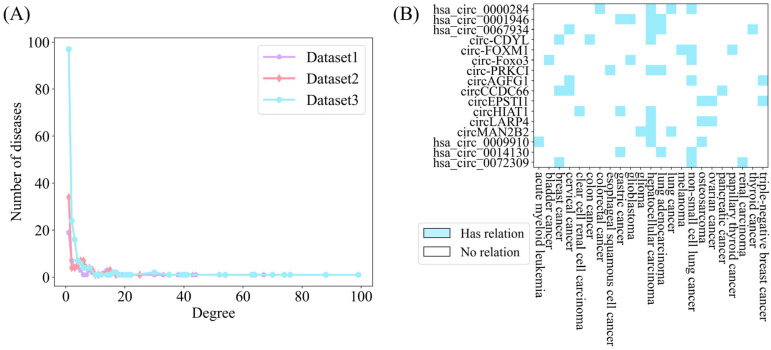
Association between CircRNA and diseases. (**A**) The horizontal axis indicates the circRNA count, while the vertical axis shows the disease count per degree. (**B**) Sparsity of some circRNA–disease associations.

Furthermore, the circRNA–disease associations are shown in Figure 3. It can be concluded that approximately 85% of the diseases are associated with no more than 10 circRNAs. Consequently, the degree of circRNA–disease associations in the three datasets is highly sparse.

### 2.2. Methods

This part provides the details of the proposed GIT-DSP model. First, the heterogeneous knowledge graph is created to enrich association information. Next, the association information is captured by the Graph Isomorphism Transformer (GIT), which consists of Transformer-based knowledge representation and Graph-Isomorphism-based information propagation and aggregation. Finally, we present the design and functionality of the Dual-Stream Neural Predictor (DSP) to predict the affinity score for CDAs.

#### 2.2.1. Heterogeneous Knowledge Graph

The explosive growth of data has enriched knowledge but also posed challenges for analysis and discovery [33]. Knowledge graphs, with their strong representation capabilities, have been widely applied in language understanding [34], reasoning [35], and recommendation systems [36]. They provide a flexible framework for integrating and analyzing large-scale biomedical data and have proven effective in generating new knowledge, uncovering higher-order associations (referring to associations between circRNAs and diseases that involve indirect connections through multiple intermediate variables or paths), and identifying unknown relationships [37,38,39]. Inspired by these findings, this study employs knowledge graph technology for CDA prediction.

**Figure 4:**
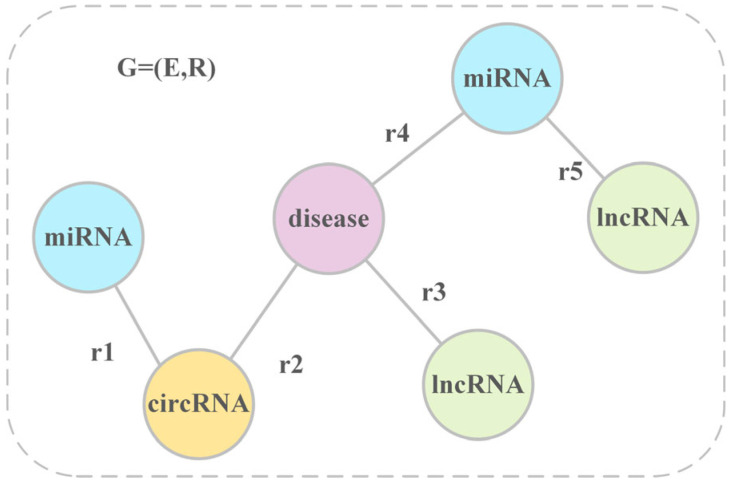
Overview of the knowledge graph. It encompasses four types of entities: disease, circRNA, lncRNA, and miRNA, as well as five different types of relationships: miRNA–circRNA, miRNA–lncRNA, disease–miRNA, disease–lncRNA, and circRNA–disease.

In this study, we first constructed three multi-source heterogeneous knowledge graphs using the three datasets organized in Section 2.1, so as to conduct experiments on each of the three datasets separately. Figure 4 provides an overview of the graphs. The ncRNA-disease knowledge graph is expressed as G=(E, R), where E is the entity collection and R is the relation collection. The intrinsic form of the knowledge graph is denoted as the (h,r,t) triplet, where h,t∈E, h denotes the head entity, r∈R denotes the relationship, and t denotes the tail entity.

Due to the nature of knowledge graphs, constructing a knowledge graph not only captures the original triples $\left(h_{1},r,h_{2}\right)$ but also acquires knowledge about the inverse relations $\left(h_{2},r^{\prime },h_{1}\right)$ and self-relations $\left(h_{1},r^{\prime \prime },h_{1}\right)$. Here, $r^{\prime }$ represents the inverse relation of r, and $r^{\prime \prime }$ represents the self-relation of r. This construction method alleviates the data sparsity problem in CDA prediction tasks to some extent and helps the model extract potential relational information from the knowledge graph.

#### 2.2.2. Transformer-Based Knowledge Representation

Knowledge representation learning (KRL) is capable of modeling complex relational graphs and generating reliable representations of entities and relationships to support subsequent tasks [40,41]. Furthermore, considering the impressive performance of Transformers in various domains, such as large language modeling [42] and multi-modal fusion [43], researchers are actively investigating their integration into the KRL domain [44,45,46]. Inspired by the explorations of these researchers, we introduce a new Transformer-encoder-based architecture for encoding entities and relations to achieve high-quality embedding representations. Specifically, let $e_{h}$, $e_{r}$, and $e_{t}$ represent the embeddings for the head entity, relation, and tail entity, respectively, assuming that all embeddings share the same dimension d. Subsequently, we concatenate the embedding vectors of the head entity h and the relation r to form the input sequence $\left[e_{h},e_{r}\right]$. Then, the Transformer encoder processes the input sequence $\left[e_{h},e_{r}\right]$ through the multi-head self-attention mechanism. Consequently, the representation of relation r is enhanced and incorporates information related to the head entity h, enabling a better capture of the complex interactions and dependencies within the sequence. The process of obtaining rich embeddings using multi-head attention is as follows:

Firstly, the query (Q), key (K), and value (V) for each header are computed by linear transformation:

(1) $$Q_{i}=W_{Q_{i}}Q,\;\;\;K_{i}=W_{K_{i}}K,\;\;\;V_{i}=W_{V_{i}}V,$$

where $W_{Q_{i}}$, $W_{K_{i}}$, and $W_{V_{i}}$ are the projection weights of the ith head, respectively.

Attention scores are then computed for each head using the scaled dot product attention mechanism:

(2) $$A_{i}\left(Q,K,V\right)=softmax\left(\frac{Q_{i}K_{i}^{T}}{\sqrt{d}}\right)V_{i}$$

The final output is obtained as follows:

(3) $$Multihead\left(Q,K,V\right)=LayerNorm(x+\left[A_{1},A_{2},\cdots ,A_{i}\right]W_{o}),$$

where LayerNorm is the layer normalization function, [·] denotes concatenation, $W_{o}$ represents the projection weight matrix, and x represents the query tensor input to the multi-head attention.

In the computation of multi-head attention in Equation (3), the attention results from multiple heads are concatenated into a larger vector. Subsequently, the final output of the multi-head attention is obtained by multiplying with the matrix $W_{o}$. At this point, the generated output not only contains information from different heads but also retains the semantic information captured by each head from different subspaces. Then, the output of the multi-head attention is passed as input x to the subsequent Feed-Forward Network (FFN):

(4) $$FFN(x)=\mathrm{LayerNorm}(x+\max\left(xW_{1}+b_{1},0\right)W_{2}+b_{2}),$$

where $b_{1}$ and $b_{2}$ represent the corresponding biases, $W_{1}$ and $W_{2}$ denote the weights, and x results from the multi-head attention.

Following the above steps, we obtained embeddings for entities and relationships. In evaluating the validity of triples (h,r,t), previous work has employed a simple dot product operation as the scoring function [47]. However, this approach fails to capture the complex associations between entities and relationships. Therefore, we designed relational matrix multiplication to enhance the expressive power of the model:

(5) $$f\left(h,r,t\right)=w_{r}\left({\hat{e}}_{r}^{T}e_{t}\right),$$

where $w_{r}$ is the relation transformation matrix, ${\hat{e}}_{r}$ is the encoded relation update embedding, and $e_{t}$ is the initial embedding of the tail entity.

Please note that ${\hat{e}}_{r}$ of Equation (5) already contains information related to the head entity h after being processed by the multi-head self-attention mechanism.

Then, the BPR loss function is applied to train the knowledge representation learning part. Compared to the traditional pairwise-ranking-based loss function [48], the BPR loss calculation method can enhance the reliability of the model and better learn the associations between entities. This is achieved by using the log-sigmoid function, which allows all samples to contribute to the gradient, unlike traditional methods that only compute gradients for samples violating ranking constraints. Additionally, BPR focuses on the relative order of scores, making it more robust to noise and outliers, and effectively handles implicit feedback where missing associations do not imply negative ones:

(6) $$L_{1}=-\sum_{\left(h,r,t,h^{\prime },t^{\prime }\right)\in O}ln\sigma \left(\hat{f}\left(h,r,t\right)-\hat{f}\left(h^{\prime },r,t^{\prime }\right)\right),$$

where $O=\{(h,r,t,h^{\prime },t^{\prime })|(h,r,t)\in R^{+},(h^{\prime },r,t^{\prime })\in R^{-}\}$ stands for the training set. $R^{+}$ is the positive association set, $R^{-}$ corresponds to the set of negative associations, σ(·) represents the sigmoid function, $\hat{f}\left(h,r,t\right)$ denotes the predictive scores of the model for the positive sample $\left(h,r,t\right)$, and $\hat{f}\left(h^{\prime },r,t^{\prime }\right)$ stands for the model’s prediction score for $\left(h^{\prime },r,t^{\prime }\right)$.

In addition, to mitigate noise in the negative samples and enhance the model’s ability to capture true associations, we employed a similarity-based negative sampling approach. Consequently, GIT-DSP is able to effectively capture both local and global patterns from complex graph-structured data, generating enriched and accurate inputs for downstream tasks.

#### 2.2.3. Graph Isomorphism Layers

In knowledge graph structures, traditional information propagation methods treat all nodes as equally important, which is unsuitable for CDA prediction tasks. To better distinguish differences between the diverse nodes, we improve upon the attention-based information propagation architecture based on previous work [21,36]. This architecture comprises two components: information propagation and information aggregation. Below is a description of a single layer of this architecture.

- Information propagation

The effective use of information from entities (e.g., circRNA, disease) is key to ensuring that high-quality expression is obtained [49,50]. In knowledge graphs, associations between entities can be obtained not only from directly connected edges but also from transitive, indirect connections. Additionally, it is important to recognize that the neighborhood information of entities has varying degrees of impact on their importance [51].

Specifically, for a node h, its corresponding set of all neighboring nodes and their relation triples is represented as $N_{h}=\{(h,r,t)|h,t\in E,r\in R\}$ [52]. To characterize entities, the ego-network surrounding node h is described as follows:

(7) $$e_{N_{h}}=\sum_{(h,r,t)\in N_{h}}\theta \left(h,r,t\right)e_{t},$$

where θ(h,r,t) represents the propagation factor determining the amount of information propagated from neighbor node t to node h.

Due to varying importance among different neighbor nodes, we employ knowledge-aware attention to compute the propagation factor for each neighbor node, with the calculation formula as follows:

(8) $$\theta \left(h,r,t\right)={(W}_{r}e_{t}{)}^{T}\tanh\left(W_{r}e_{h}+e_{r}\right),$$

where $W_{r}$ serves as the transformation matrix, mapping entities to the relation space, and tanh denotes the non-linear activation function.

Furthermore, to better understand information propagation in the network and enhance focus on important nodes, it is necessary to normalize the propagation factors mentioned above:

(9) $$\theta \left(h,r,t\right)=\frac{exp(\theta \left(h,r,t\right))}{\sum_{(h,r^{\prime },t^{\prime })\in N_{h}}exp(\theta (h,r^{\prime },t^{\prime }))}$$

After the above processing, GIT-DSP can effectively distinguish the feature representations of nodes. Additionally, normalization during attention propagation helps reduce noise influence, allowing the attention to focus more on important nodes.

- Information aggregation

**Figure 5:**
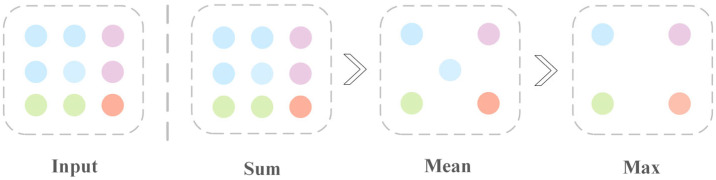
Ranking of the expressive power of Sum, Mean, and Max on multisets. The input part represents the aggregated neighborhood network; Sum learns the entire multiset, Mean learns the overall distribution, and Max ignores redundant information.

In graph neural networks, injectivity (one-to-one mapping) is a key indicator for evaluating aggregation capability. However, the traditional average aggregation method can maintain the consistency of feature scales to a certain extent, but it may ignore some detail information compared to the summation aggregation, while the maximum aggregation, although it can highlight the most important features, may also lead to the loss of some useful neighborhood information. In contrast, the summation method preserves the complete information of neighboring nodes and meets the definition of injectivity. Figure 5 demonstrates the information aggregation capabilities of the three strategies mentioned above.

**Figure 6:**
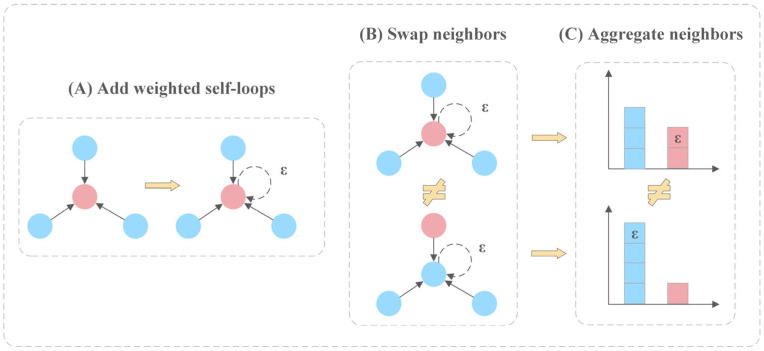
The principle of graph isomorphism network. (**A**) Weighted self-loops are added to the nodes to ensure they retain their unique feature information during aggregation. (**B**) Exchange the neighboring nodes with weighted self-loops. (**C**) The corresponding parts from (**B**) are aggregated to update each node’s representation.

In circRNA–disease association prediction, capturing both local relationships between circRNAs and diseases and global graph patterns is critical. Inspired by the Weisfeiler–Lehman (WL) graph isomorphism test, the Graph Isomorphism Network (GIN) [53] enhances the discriminative power of graph neural networks by ensuring that the node features remain distinguishable and are progressively updated through weighted self-loops (Figure 6). Specifically, the process starts with adding weighted self-loops to each node (Figure 6A), ensuring the nodes retain their unique features during aggregation. Subsequently, neighboring node features are swapped (Figure 6B), enhancing the model’s ability to capture local graph structures and distinguish nodes with similar local but different global positions. Finally, an injective aggregation of neighboring node features updates each node’s representation (Figure 6C), reliably capturing local and global graph features. Compared to other GNN models like Graph Convolutional Networks (GCNs) and Graph Attention Networks (GATs), GIN effectively avoids over-smoothing in deeper networks and employs injective neighborhood aggregation to reliably capture local and global graph features.

In summary, the GIN architecture is an effective means of capturing the intricate local associations between circRNAs and diseases as well as the broader global structure of the graph. Thus, GIN is an ideal method for predicting non-linear and heterogeneous circRNA–disease associations. After multiple layers of neighborhood aggregation and updating, GIN is capable of progressively capturing higher-order features within the graph and reflecting the global graph structure through continuous node feature updating. The GIN convolution with a sum aggregation approach not only ensures the learning of the entire network structure but also enhances the model’s ability to represent the graph structure. The aggregation of entity h and its ego-network is defined as follows:

(10) $$f_{GIN}^{\left(l\right)}\left(e_{h}^{\left(l-1\right)},e_{N_{h}}^{\left(l-1\right)}\right)=LeakyReLU\left({MLP}^{\left(l\right)}\left(\left(1+\epsilon^{\left(l\right)}\right)\cdot e_{h}^{\left(l-1\right)}+{AGG}_{N_{h}}^{\left(l-1\right)}\right)\right),$$

(11) $${AGG}_{N_{h}}^{\left(l-1\right)}=\sum_{j\in N_{h}}e_{j}^{\left(l-1\right)},$$

where LeakyReLU(·) denotes the activation function, l represents the layers, ϵ denotes the learnable parameter, and MLP stands for multi-layer perceptron. Equation (11) is the cumulative implementation for neighboring nodes.

To evaluate the effectiveness of the GIN aggregation strategy, the GraphSage aggregator [54], Bi-Interaction aggregator [36], and the currently best-performing GCN aggregator [55] were set as comparisons.

(12) $$f_{GraphSage}^{\left(l\right)}\left(e_{h}^{\left(l-1\right)},e_{N_{h}}^{\left(l-1\right)}\right)=LeakyReLU(W(e_{h}^{\left(l-1\right)}||e_{N_{h}}^{\left(l-1\right)})),$$

(13) $$f_{Bi-Interaction}^{\left(l\right)}\left(e_{h}^{\left(l-1\right)},e_{N_{h}}^{\left(l-1\right)}\right)=LeakyReLU\left(W\left(e_{h}^{\left(l-1\right)}+e_{N_{h}}^{\left(l-1\right)}\right)\right)+LeakyReLU\left(W\left(e_{h}^{\left(l-1\right)}⊙e_{N_{h}}^{\left(l-1\right)}\right)\right),$$

(14) $$f_{GCN}^{\left(l\right)}\left(e_{h}^{\left(l-1\right)},e_{N_{h}}^{\left(l-1\right)}\right)=LeakyReLU(W(e_{h}^{\left(l-1\right)}+e_{N_{h}}^{\left(l-1\right)})),$$

where $f_{GraphSage}^{\left(l\right)}(\cdot )$, $f_{Bi-Interaction}^{\left(l\right)}\left(\cdot \right)$, and $f_{GCN}^{\left(l\right)}\left(\cdot \right)$ denote the node update functions of the GraphSage aggregator, the Bi-Interaction aggregator, and the GCN aggregator at the lth layer, respectively; || denotes concatenation; and ⊙ represents element-wise multiplication.

Therefore, we further extend the formula to describe the embedding of node h at layer l. Specifically, the embedding of node h at layer l can be iteratively updated as $e_{h}^{\left(l\right)}$:

(15) $$e_{h}^{\left(l\right)}=\varphi \left(\left(1+\epsilon^{\left(l-1\right)}\right)\cdot e_{h}^{\left(l-1\right)}+f(e_{N_{h}}^{\left(l-1\right)})\right)$$

The information propagated by node h within the l-ego network is defined as:

(16) $$e_{N_{h}}^{\left(l-1\right)}=\sum_{(h,r,t)\in N_{h}}\theta \left(h,r,t\right)e_{t}^{\left(l-1\right)},$$

where φ(·) denotes an injective function and $f\left(\cdot \right)$ represents an operation on a multiset.

As the number of stacked layers increases, we can obtain more reliable and enriched coding information, thus better describing the multi-hop neighbor association information of circRNA–disease association. Based on previous research [19,36], we configured the layers l to 4, as four-order information is sufficient to capture the latent features and associations in the knowledge graph without causing over-smoothing, thus achieving a more accurate representation.

#### 2.2.4. Dual-Stream Neural Predictor

After multiple layers of information propagation and aggregation, a high-quality embedded representation of each entity at each layer is obtained. Then, similar to previous studies [17], the information in each layer is concatenated to enrich the final node representation:

(17) $$e_{c_{i}}=e_{i}^{\left(0\right)}||\cdots ||e_{i}^{\left(l\right)}\;,\;e_{d_{j}}=e_{j}^{\left(0\right)}||\cdots ||e_{j}^{\left(l\right)},$$

where || denotes concatenation, $e_{c_{i}}$ indicates the circRNA embedding in layer l, and $e_{d_{j}}$ stands for the disease embedding in layer l.

Based on the above approaches, we obtained rich node representations. To capture the complex relationships more accurately between nodes, we define $e_{c_{i}}||e_{d_{j}}$ as the embedding of CDAs. Next, we designed a Dual-Stream Neural Predictor to perform the calculations, thereby enhancing the model’s non-linear prediction capabilities.

In the DSP module, we designed Stream 1 as a deeper network and Stream 2 as a shallower but wider network, based on the observation that deeper networks can capture more global and abstract features, while shallower networks are better suited to capturing local features in data. The structure of Stream 1 is a progressively deeper network designed to iteratively extract and compress features. At each layer, the feature dimensions are reduced by half, enabling the network to learn multiple levels of data abstraction and uncover deep non-linear associations. To balance this, Stream 2 adopts a shallower but broader architecture, which captures key features quickly and mitigates the risk of overfitting. This design ensures a comprehensive exploration of linear relationships while still providing a degree of abstraction through its moderate depth. The outputs of these two data streams are combined to form a unified representation, enabling the model to comprehensively explore multi-source heterogeneous knowledge. This combined representation ensures both the stability and reliability of the predictions.

The specific calculation process is as follows:

(18) $$\hat{y}\left(i,j\right)=\sigma_{l}W_{l}\left({\sigma_{l-1}W}_{l-1}\left(\cdots \left(W_{1}\left(\delta \right)+b_{1}\right)\cdots \right)+b_{l-1}\right)+b_{l},$$

(19) $$\delta ={MLP}_{1}(e_{c_{i}}||e_{d_{j}})||{MLP}_{2}(e_{c_{i}}||e_{d_{j}})),$$

where $\sigma_{l}$, $W_{l}$, and $b_{l}$ represent the activation function, learnable parameter, and bias term of layer l, respectively, and ${MLP}_{1}$ and ${MLP}_{2}$ represent Stream 1 and Stream 2, respectively.

Next, the BPR loss function is introduced to optimize the above results:

(20) $$L_{2}=-\sum_{\left(i,j,j^{\prime }\right)\in P}ln\sigma \left(\hat{y}\left(i,j\right)-\hat{y}\left(i,j^{\prime }\right)\right),$$

where P represents the set containing unobserved positive and negative triplet samples in equal proportions.

Finally, to realize end-to-end optimization, we define the total loss function as follows:

(21) $$L=L_{1}+L_{2}+{\lambda ||\alpha ||}^{2},$$

where α denotes the parameters, while λ is the regularization parameter.

In this section, we introduced our methodology, including the use of knowledge graph technology, knowledge embedding representation, attention-based information propagation, and aggregation, as well as the design of the prediction module. Each part was discussed in detail. The integrated application of these methods forms the backbone of the CDA prediction model. By designing the Dual-Stream Neural Predictor, the GIT-DSP model has stronger non-linear prediction capabilities. Therefore, we believe that GIT-DSP is now capable of obtaining rich feature representations between nodes and accurately capturing the complex associations between them. Next, we will validate the effectiveness of GIT-DSP with specific experiments.

## 3. Results and Discussion

### 3.1. Evaluation Metrics

To showcase the superiority of GIT-DSP, we conducted systematic experiments employing fivefold cross-validation (CV) and compared it to other SOTAs. In the fivefold CV, the dataset is split into five equal sections. Four sections are selected for training, and the other one serves as testing, rotating each part as the test set to ensure comprehensive learning from the data. Then, the result was taken using the mean fivefold CV. The performance is evaluated using accuracy (Acc), recall (Rec), precision (Pre), and F1 score (F1) as follows:

(22) $$Acc=\frac{TP+TN}{TP+TN+FP+FN},$$

(23) $$Rec=\frac{TP}{TP+FN},$$

(24) $$Pre=\frac{TP}{TP+FP},$$

(25) $$F1=\frac{2\cdot Pre\cdot Rec}{Pre+Rec},$$

where TP and TN denote the correctly identified positive and negative samples, while FP and FN represent incorrectly identified positive and negative samples.

Additionally, the area under the Precision–Recall (PR) curve (AUPR) and the area under the Receiver Operating Characteristic (ROC) curve (AUC) are used to evaluate the model comprehensively. The PR curve reflects the model sensitivity, while ROC shows the relationship of the true positive rate (TPR) to the false positive rate (FPR). The mathematical expressions for TPR and FPR are as follows:

(26) $$TPR=\frac{TP}{TP+FN},$$

(27) $$FPR=\frac{FP}{FP+TN}$$

To further measure the model prediction ability, we also included the number of correctly identified CDAs as one of the model evaluation criteria. We use Top−K to indicate the count of associations between the top K similar RNAs from model predictions and the related diseases.

### 3.2. Parameter Setting

The model is implemented on NVIDIA V100 GPUs using the PyTorch version 2.0.0, with parameter settings derived from extensive comparative experiments.

In the training phase, we used 100 epochs and a learning rate of $1\times 10^{-4}$ to adequately train the model. To accelerate convergence and improve model stability, we set the dynamic learning rate decay based on model performance scheduling and optimized it using the Adam optimizer. Furthermore, to fully capture the higher-order association information, we chose a four-layer propagation structure [512-256-128-64], with the hidden layer set to 256 and both entity and relation embeddings set to 2048. Additionally, we used a 0.1 dropout rate to mitigate overfitting. In the learning phase of the knowledge representation, there are 16 attention heads, and a dropout of 0.2 was applied to each KRL module. For the GIN aggregation strategy, we chose the sum method, which showed the best performance.

In the prediction phase, we set 32 epochs, a $5\times 10^{-4}$ learning rate, and a $1\times 10^{-7}$ weight decay to reduce overfitting. The Stream 1 structure employs a gradually decreasing strategy [6016, 3008, 1504, 752], which helps the network learn the data at different levels of abstraction. The Stream 2 structure is designed as [6016, 6016, 3008], which aims to capture data features and identify different pattern associations from multiple perspectives. Additionally, GIT-DSP achieved optimal performance with a 1:10 positive-to-negative sample ratio and the seven most similar circRNAs.

### 3.3. Performance Comparison

To highlight the advantages of GIT-DSP, we compared it with nine representative SOTAs, which cover three mainstream categories: information-propagation-based methods (KATZHCDA [7], RWR [9], and CD-LNLP [11]), traditional machine-learning-based methods (RWR-KNN [14], ICIRCDA [15], and RNMFLP [16]), and deep-learning-based methods (DMFCDA [17], GMNN2CD [20], and KGETCDA [21]). The advantages and disadvantages of the comparison models are presented in Table 2.

The detailed methodologies of these nine comparison models are outlined below, offering a comprehensive overview of their operational mechanisms in predicting circRNA–disease associations.

- KATZHCDA: Uses information from heterogeneous graphs to predict associations between circRNA and diseases through the KATZ algorithm.
- RWR: A restart random wander method is used to simulate the process of random wandering on the network, and the restart probability is introduced to regulate the direction of wandering, which achieves the prediction of potential CDAs.
- CD-LNLP: A linear neighborhood label propagation approach that uses known association graphs to propagate labels and thus predict CDAs.
- RWR-KNN: A method that combines the RWR algorithm and the K-nearest neighbor algorithm, with the former evaluating node similarity and the latter enhancing node classification accuracy to improve CDA prediction.
- ICIRCDA: Calculates initial circRNA–disease associations based on diverse biological information, corrects false-negative associations through local association profiles, and uses matrix factorization to compute the final CDA scores.
- RNMFLP: Uses robust non-negative matrix factorization to capture potential CDA pairs and employs the LP algorithm to enhance CDA prediction accuracy from the candidate association pairs.
- DMFCDA: Models non-linear associations through deep matrix factorization and multi-layer neural networks, automatically learning the potential representations of circRNA–disease associations.
- GMNN2CD: Uses graph Markov neural networks to obtain deep features from low-dimensional representations and propagates labels with a graph autoencoder to predict CDAs.
- KGETCDA: Uses a Transformer for knowledge representation and a multi-layer perceptron to calculate CDA affinity scores from embeddings for prediction.

Next, specific comparison experiments were conducted using the same device, with the parameters for each comparison model being the optimal ones for each model.

**Figure 7:**
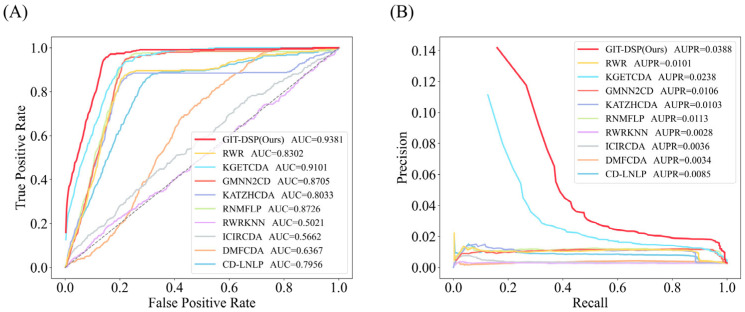
Performance comparison of AUC (**A**) and AUPR (**B**) on Dataset 1. The compared models include information-propagation-based models (KATZHCDA, RWR, and CD-LNLP), traditional machine learning models (RWR-KNN, ICIRCDA, and RNMFLP), and deep learning models (DMFCDA, GMNN2CD, and KGETCDA).

For Dataset 1, as shown in Figure 7, GIT-DSP achieved an average AUC of 0.9381, outperforming the best benchmark by 3.08%. This improvement is mainly due to the GIN aggregator’s ability to capture both local and global associations in the knowledge graph, preserving node-level distinctions and avoiding the over-smoothing problem found in models such as GCN. The DSP further enhances the model’s ability to capture complex, non-linear relationships, particularly in sparse data. In terms of AUPR, GIT-DSP outperformed the benchmark by 63.03%, demonstrating its strength in handling unbalanced datasets and extracting hidden associations, which are critical for CDA prediction.

**Figure 8:**
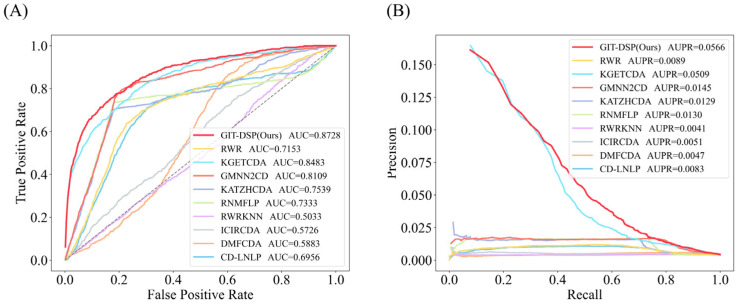
Performance comparison of AUC (**A**) and AUPR (**B**) on Dataset 2. The compared models include information-propagation-based models (KATZHCDA, RWR, and CD-LNLP), traditional machine learning models (RWR-KNN, ICIRCDA, and RNMFLP), and deep learning models (DMFCDA, GMNN2CD, and KGETCDA).

For Dataset 2, as shown in Figure 8, GIT-DSP achieved an average AUC of 0.8728, surpassing the best baseline by 2.89%. This improvement reflects the flexibility of the GIN aggregator to capture both first-order and higher-order associations, even in sparse, complex data. While traditional models such as GraphSage and Bi-Interaction struggle to generalize, the DSP module effectively models both linear and non-linear relationships, improving adaptability. GIT-DSP’s average AUPR of 0.0566, an increase of 11.20%, further highlights its ability to maintain precision and recall in unbalanced datasets.

**Figure 9:**
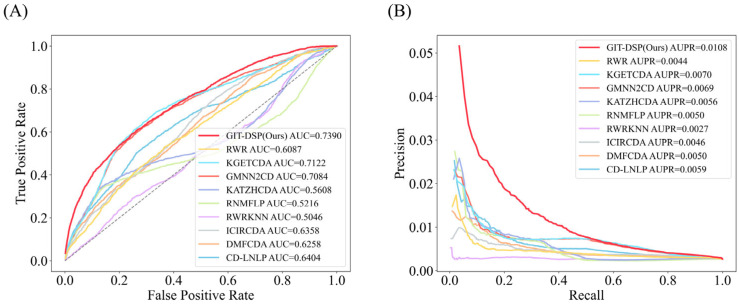
Performance comparison of AUC (**A**) and AUPR (**B**) on Dataset 3. The compared models include information-propagation-based models (KATZHCDA, RWR, and CD-LNLP), traditional machine learning models (RWR-KNN, ICIRCDA, and RNMFLP), and deep learning models (DMFCDA, GMNN2CD, and KGETCDA).

For Dataset 3, as shown in Figure 9, GIT-DSP achieved an average AUC of 0.7390, outperforming the best benchmark by 3.76%. This improvement demonstrates the robustness of the GIN aggregator in capturing complex associations and effectively managing the extreme sparsity and heterogeneity present in ultra-large datasets. The model’s ability to preserve meaningful distinctions between graph nodes ensures superior handling of intricate multi-source relationships. In terms of AUPR, GIT-DSP achieved 0.0108, a significant increase of 54.29% over the best baseline. This highlights the model’s ability to maintain high recall and precision rates, particularly when identifying critical but subtle circRNA–disease associations in highly unbalanced datasets. The inclusion of the DSP further enhances the model’s adaptability by effectively addressing non-linear association patterns, which are critical for accurate prediction in this challenging dataset.

The detailed performance comparison results for all models across three datasets are presented in Table 3. As shown, the GIT-DSP model consistently outperforms all other models in terms of AUC and AUPR across all datasets. Specifically, the highest AUC and AUPR values are highlighted in bold, further reinforcing the model’s exceptional ability in predicting circRNA–disease associations. Compared with the best baseline, GIT-DSP achieves a 3.08% and 63.03% improvement in AUC and AUPR, respectively, on Dataset 1. For Dataset 2, it surpasses the best baseline by 2.89% in AUC and 11.20% in AUPR. On the most challenging Dataset 3, GIT-DSP achieves a 3.76% increase in AUC and an impressive 54.29% improvement in AUPR. These results demonstrate the model’s robustness in handling sparse and highly heterogeneous data.

**Figure 10:**
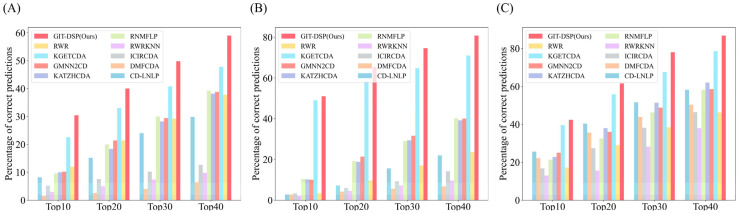
Comparison of the average number of accurately identified associations for Dataset 1 (**A**), Dataset 2 (**B**), and Dataset 3 (**C**). The compared models include information-propagation-based models (KATZHCDA, RWR, and CD-LNLP), traditional machine learning models (RWR-KNN, ICIRCDA, and RNMFLP), and deep learning models (DMFCDA, GMNN2CD, and KGETCDA).

Additionally, we generated a bar graph illustrating the number of correctly predicted CDAs by GIT-DSP, as shown in Figure 10. The average number of accurately identified associations from the Top 10 to Top 40 in Dataset 1 is 30.4, 40.0, 49.8, and 59.0, respectively; in Dataset 2, the corresponding numbers are 51.0, 65.4, 74.6, and 80.8; and in Dataset 3, the number of associations were 42.4, 61.6, 78.0, and 86.8, respectively. These results underscore GIT-DSP’s superior capacity to detect and prioritize true associations across datasets of varying density, especially at higher cutoff points (Top 40). This demonstrates not only its effectiveness in capturing the most prominent associations but also its ability to uncover more subtle and complex relationships that other models often miss. Notably, in Dataset 3, where data sparsity presents a significant challenge, GIT-DSP maintains strong performance in identifying correct associations, underscoring its robustness in handling real-world biological data characterized by intricate multi-relational structures. The ability of GIT-DSP to generalize and maintain accuracy across different datasets highlights its adaptability, especially when compared to models that tend to overfit or struggle under similar conditions.

Overall, the performance of other methods is typically limited in complex multi-relationship scenarios and when handling sparse data. Many existing models, such as GCN, Bi-Interaction, and GraphSage, fail to capture the full range of relational information due to their inherent limitations in either aggregation power or feature extraction. GCN tends to over-smooth node representations, making it difficult to distinguish between nodes of different categories, especially in deep layers. Bi-Interaction and GraphSage, on the other hand, lack the expressiveness needed to fully capture the intricate non-linear relationships in biological networks. These limitations become apparent in highly sparse data settings, where hidden associations between entities are harder to discover without a robust, multifaceted approach such as that provided by GIT-DSP.

By leveraging knowledge graph techniques in conjunction with the GIN aggregator and DSP, our approach ensures that both the structural information from the graph and the non-linear association patterns are effectively exploited. This synergy results in high-quality embeddings and rich knowledge representations, enabling GIT-DSP to outperform baseline models even under the challenging conditions of multi-relational and sparse data. Due to its generalization ability, we believe that GIT-DSP has great potential not only in CDA prediction but also in other domains, such as protein–gene and ncRNA–gene association prediction.

### 3.4. Ablation Study

In this part, we conduct ablation studies. Notably, to extensively evaluate the performance and stability of GIT-DSP, all the ablation experiments were performed under a five-fold cross-validation scenario, with only one module being replaced at a time. Keeping other settings unchanged, we first examined the impact of different aggregation modules in KRL (GIN (Ours), GCN, GraphSage, and Bi-Interaction) and different prediction modules (DSP (Ours), SMLP, and MLP) on model performance, where DSP denotes our predictor and SMLP represents stacking two MLPs with the same structure. We then proceeded to investigate the effect of different methods for computing neighborhood features (Max, Mean, and SUM) in GIN. To be more rigorous, the effect of different attention heads and layers in KRL on the model is also studied.

#### 3.4.1. Effect of Different Aggregation and Prediction Modules

**Figure 11:**
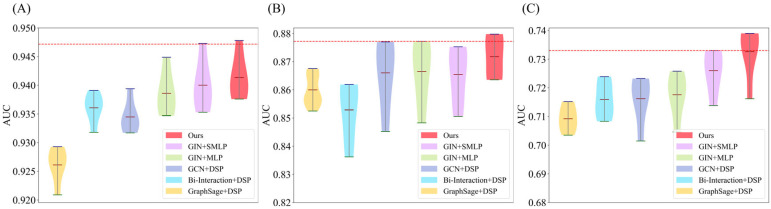
Violin plots comparing the performance of different aggregation and prediction modules. (**A**) AUC values for Dataset 1; (**B**) AUC values for Dataset 2; (**C**) AUC values for Dataset 3. It covers GIN+MLP, GIN+SMLP, and GIN+DSP(Ours) based on GIN, GCN+DSP based on GCN, BI-Interaction+DSP based on Bi-Interaction, and GraphSage+DSP based on GraphSage.

In this section, we remove and replace the aggregation and prediction modules to comprehensively showcase the effectiveness of our approach. Specifically, the design of the prediction module was first carried out based on GIN, and different MLP configurations were tried. Then, a conventional single-layer MLP, called GIN+MLP, was used as a benchmark, and two MLP stacks with the same structure (GIN+SMLP) and two MLP stacks with different structures (GIN+DSP(Ours)) were used, respectively. Similarly, experiments with the above configurations based on GCN, Bi-Interaction, and GraphSage were conducted to fully compare the effects of different aggregation modules and different prediction modules on model performance. Figure 11 illustrates the fivefold CV results.

Based on the results of the three datasets, our method demonstrates significantly higher AUC values compared to the other three aggregation modules. This performance advantage likely stems from GIN’s ability to retain more expressive neighborhood features during aggregation, enabling it to capture both local and global information in the graph structure more effectively. GIN’s injective nature allows it to distinguish between different node neighborhoods more precisely than GCN, Bi-Interaction, or GraphSage, which may explain its superior performance. GCN, for instance, tends to over-smooth, making it increasingly difficult to differentiate between nodes of different classes after several layers, while Bi-Interaction and GraphSage may struggle to capture the complex topological relationships critical for accurate CDA prediction.

Further analysis of prediction methods shows that GIN+DSP stands out among all prediction modules. The DSP, which models both linear and non-linear associations within the data, contributes significantly to this improvement. By capturing different association patterns through its dual streams, DSP outperforms single-stream predictors such as SMLP and standard MLP, which struggle with the complexity of graph structures and the non-linearity of biological data. This dual-stream design helps to reduce overfitting and improves generalization, especially when combined with the powerful feature aggregation capabilities of GIN.

#### 3.4.2. Effect of Different Neighborhood Feature Calculations

**Figure 12:**
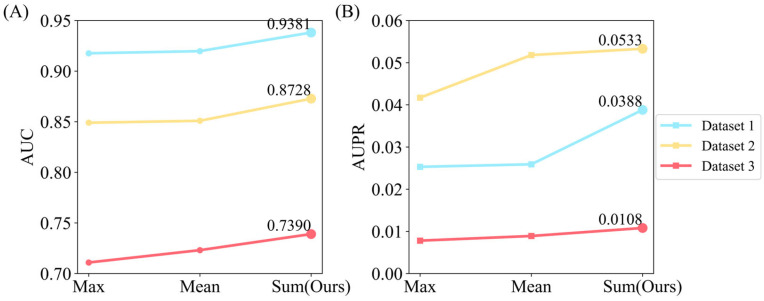
Comparison of methods for neighborhood feature calculation. (**A**,**B**) represent the AUC and AUPR values on the three datasets, respectively.

We further explored the effects of the Max, Mean, and Sum aggregation strategies in the Graph Isomorphism Network (GIN) on model performance. Figure 12 illustrates the changes in AUC and AUPR values when replacing the GIN neighborhood aggregation strategy with these three strategies sequentially in all datasets.

The line chart shows that the Sum strategy has superior AUC and AUPR to the Mean and Max strategies on all datasets. The reason for this may lie in the ability of the Sum strategy to retain more comprehensive information from node neighborhoods, as it does not discard important features during aggregation. The Max aggregation only retains the most prominent feature, potentially leading to loss of information, while the Mean strategy averages out features, reducing the distinction between nodes. Consequently, the Sum aggregation method effectively combines all feature information, providing a more comprehensive representation of the node’s local environment, which is crucial for capturing the complex associations necessary for accurate CDA prediction.

#### 3.4.3. Effect of Different Attention Heads and Layers

**Figure 13:**
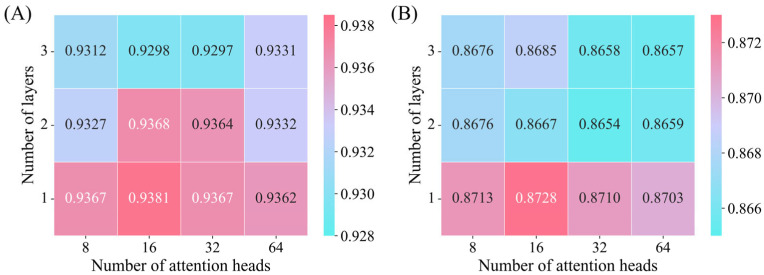
Performance comparison of different attention heads and layers. (**A**) AUC heat map for Dataset 1; (**B**) AUC heat map for Dataset 2.

To thoroughly study the effectiveness of Transformer-based knowledge representation learning, we designed ablation experiments with different attention heads and layers. Note that increasing the number of attention heads and layers raises the time and space complexity. Therefore, based on the experience from previous related studies [19,21], we set the number of attention heads to 8, 16, 32, and 64 and the number of layers to 1, 2, and 3. With these configurations, we systematically evaluated the effect of varying attention heads and layers on GIT-DSP. Figure 13 shows the experimental results.

Experimental results show that our model achieves the best performance when the number of attention heads is set to 16, and the number of encoder layers is set to 1. The use of 16 attention heads allows the model to focus on multiple aspects of the graph’s structure simultaneously, capturing diverse association patterns between nodes. However, using more than 16 heads introduces redundancy and noise, as the additional heads may attend to less informative or irrelevant aspects of the graph [56]. Similarly, increasing the number of layers beyond 1 does not yield better performance. While deeper layers may theoretically capture higher-order associations, they also introduce the risk of overfitting and noise accumulation, particularly in a complex, dense graph like the one used in CDA prediction. Consequently, the balanced architecture of 16 attention heads and 1 layer of encoders in our model represents an optimal configuration for maintaining efficiency and predictive power.

### 3.5. Case Study

To examine the reliability of GIT-DSP, we performed case studies on Dataset 1 and Dataset 2. According to [57], we first utilized the GIT module of the model to learn known association information. Next, the DSP was employed to calculate the affinity scores in the circRNA–disease association matrix, which reflects the strength of associations between circRNAs and diseases. The affinity scores were computed using Equation (18). After obtaining the score matrix, we ranked all predicted scores in descending order to identify the most relevant circRNAs for each disease. This ranking process ensures the identification of the most promising circRNA–disease associations predicted by the model. Finally, we verified the results based on the predicted rankings, combined with public datasets and relevant research.

For Dataset 1, we chose acute myeloid leukemia (AML) for our case study. According to statistics, the 5-year relative survival for AML patients in the United States is merely 31.9%, accounting for 1.3% of newly diagnosed cancer cases in the country [58]. Given the challenges in treating AML, increasing research is focusing on circRNA. Effective intervention in circRNA can provide new targets and strategies for AML treatment [59]. Table 4 lists the predicted AML-related circRNAs.

According to Table 4, among the top 10 circRNAs potentially associated with acute myeloid leukemia (AML), 8 (hsa_circ_100290, circ-*ANAPC7*, circ*PAN3*, hsa_circ_0035381, hsa_circ_0001187, hsa_circ_0004277, circ_*AFF2*, and hsa_circ_0000254) have already been validated. For example, circ-*ANAPC7* indirectly regulates AML pathogenesis by adsorbing the miR-181 family [60]. Additionally, circ-*ANAPC7* is also correlated with the peripheral blood leukocyte count and bone marrow blast percentage [61]. Circ*PAN3* promotes AML drug resistance by regulating autophagy [62]. Hsa_circ_0004277 inhibits AML cell activity by sponging miR-134-5p or through overexpression [63].

For Dataset 2, we conducted a lung cancer case study. The latest statistics [64] show lung cancer comprises 12.4% of all cancer cases and significantly impact human health. Studying the expression patterns and functions of circRNAs related to lung cancer is crucial for its diagnosis and treatment. Table 5 lists the predicted lung-cancer-related circRNAs.

Table 5 shows that 7 of the top 10 candidate lung-cancer-related circRNAs (hsa_circ_0007059, circ*MTO1*, circ-*PRKCI*, hsa_circ_0046264, circ*PUM1*, *CDR1as*, and circ-*ERBB2*) are supported by research in the literature. For example, the top-ranked hsa_circ_0007059 inhibits lung cancer cell proliferation by hindering miR-378 expression [65]. The third-ranked circ*MTO1* can inhibit lung adenocarcinoma (LUAD) growth by inactivating the Notch signaling pathway through increased expression of QKI-5 [66]. Recent studies have found that the fourth-ranked circ-*PRKCI* promotes LUAD cell proliferation, while inhibiting circ-*PRKCI* expression can restrict the spread of lung cancer cells [67].

In conclusion, through case studies on acute myeloid leukemia (AML) and lung cancer, we have verified the accuracy and reliability of the GIT-DSP in predicting circRNA–disease associations. Meanwhile, this study also demonstrated its significant value in biological research and provided new insights and methods for in-depth exploration and understanding of the molecular mechanisms of diseases. It should be noted that the circRNAs that have not been validated in the prediction results warrant further exploration and validation by biologists.

## 4. Conclusions

Circular RNAs (circRNAs) have emerged as pivotal elements in biomedical research, particularly in understanding human disease mechanisms and advancing diagnosis and treatment strategies. Predicting circRNA–disease associations (CDAs) contributes to a deeper understanding of gene regulatory networks and offers valuable insights for precision medicine. However, the inherent sparsity of CDA data and the complexity of biological associations have posed significant challenges to existing computational approaches. In this paper, we proposed GIT-DSP, an innovative approach for predicting circRNA–disease associations by integrating knowledge graph techniques with a Graph Isomorphism Transformer and a Dual-Stream Neural Predictor. By incorporating diverse biological entities and associations, GIT-DSP thoroughly explores complex and diverse high-order interactions, alleviating data sparsity and achieving accurate and efficient predictions of circRNA–disease associations. Extensive experimental analyses demonstrate that GIT-DSP can effectively extract more comprehensive and insightful association information, significantly outperforming other state-of-the-art methods in the prediction of circRNA–disease associations.

Although the GIT-DSP model has demonstrated excellent performance in circRNA–disease association prediction, there is still some optimizable space for its application and development. Firstly, the incorporation of other biological association information, such as RNA sequences and proteins, can be considered for prediction to enhance model robustness. Secondly, the extension of the model to multi-biomarker prediction, such as mRNA, as well as other biological prediction scenarios, can be explored to improve the usefulness of GIT-DSP. Thirdly, while summation aggregation effectively captures node neighborhood information, model performance may drop due to data scale differences. We will explore hybrid strategies, combining summation with methods like averaging or max-pooling, to enhance robustness and generalization. Finally, while the GIT-DSP model has made significant progress in predicting circRNA–disease associations, its transformer-based architecture brings about relatively high computational complexity. This can result in substantial memory usage when handling large-scale datasets. In the future, we will make more efforts to enhance the practicality and usability of the model.

## Figures and Tables

**Table 1:** Association information of the three datasets.

Dataset	Databases	circRNA– Disease	circRNA– miRNA	miRNA– Disease	lncRNA– miRNA	lncRNA– Disease	Total
Dataset 1	Circad [22], CircRNADisease [23], LncRNASNP2 [24], LncRNADisease [25]	346	146	106	202	527	1327
Dataset 2	CircR2Cancer [26], LncRNASNP2 [24], LncRNADisease [25]	647	756	732	308	1066	3509
Dataset 3	Circad [22], MNDR [27], Lnc2Cnacer [28], LncRNADisease [29], CircRNADisease [23], Circ2Disease [30], CircR2Cancer [26], HMDD [31], StarBase [32]	1399	1129	10,154	9506	3280	25,468

**Table 2:** Overview of the nine comparison models.

Categories	Model	Advantages	Disadvantages
Information Propagation	KATZHCDA	Simple and efficient, suitable for capturing local associations.	Struggles with sparse data and global association capture.
	RWR		
	CD-LNLP		
Traditional Machine Learning	RWR-KNN	Robust to noise and leverages classic algorithms for classification.	Requires feature engineering and is computationally expensive for large data.
	ICIRCDA		
	RNMFLP		
Deep Learning	DMFCDA	Automatic extraction of higher-order features.	Difficulty in mining and distinguishing higher-order associations.
	GMNN2CD		
	KGETCDA		

**Table 3:** Performance of the model under three datasets.

Model	Dataset 1		Dataset 2		Dataset 3	
	AUC	AUPR	AUC	AUPR	AUC	AUPR
KATZHCDA	0.8033	0.0103	0.7539	0.0129	0.5608	0.0056
RWR	0.8302	0.0101	0.7153	0.0089	0.6087	0.0044
CD-LNLP	0.7956	0.0085	0.6956	0.0083	0.6404	0.0059
RWR-KNN	0.5021	0.0028	0.5033	0.0041	0.5046	0.0027
ICIRCDA	0.5662	0.0036	0.5726	0.0051	0.6358	0.0046
RNMFLP	0.8726	0.0113	0.7333	0.0130	0.5216	0.0050
DMFCDA	0.6367	0.0034	0.5883	0.0047	0.6258	0.0050
GMNN2CD	0.8705	0.0106	0.8109	0.0145	0.7084	0.0069
KGETCDA	0.9101	0.0238	0.8483	0.0509	0.7122	0.0070
GIT-DSP(Ours)	0.9381	0.0388	0.8728	0.0566	0.7390	0.0108
Improvement (%)	3.08%	63.03%	2.89%	11.20%	3.76%	54.29%

**Table 4:** Top 10 predicted candidate circRNAs for AML.

Disease	Candidate circRNA	Rank	Evidence (PMID)
acute myeloid leukemia	hsa_circ_100290	1	30424877
	hsa_circ_0000488	2	Unknown
	circ-*ANAPC7*	3	29969755, 34879367
	circ*PAN3*	4	30395908, 31401408
	hsa_circ_0035381	5	28282919, 35917008
	hsa_circ_0001187	6	28282919, 37280654
	hsa_circ_102533	7	Unknown
	hsa_circ_0004277	8	35412941
	circ_*AFF2*	9	28282919
	hsa_circ_0000254	10	29950198

**Table 5:** Top 10 predicted candidate circRNAs for lung cancer.

Disease	Candidate circRNA	Rank	Evidence (PMID)
lung cancer	hsa_circ_0007059	1	31351967
	circ-*PRMT5*	2	Unknown
	circ*MTO1*	3	30975029
	circ-*PRKCI*	4	29588350, 33155212, 33660800
	hsa_circ_0046264	5	29891014
	circ*PUM1*	6	30528736, 37326964
	hsa_circ_0003028	7	Unknown
	*CDR1as*	8	30841451, 31881486, 36508830
	hsa_circ_0007915	9	Unknown
	circ-*ERBB2*	10	31109436, 33506582

## Data Availability

All data are present within the manuscript or available upon request to the corresponding author, Haodong Zhu (2011016@zzuli.edu.cn).
